# Identification of psychiatric patients with high mortality and low medical utilization: a population-based propensity score-matched analysis

**DOI:** 10.1186/s12913-020-05089-6

**Published:** 2020-03-18

**Authors:** Jong-Yi Wang, Cheng-Chen Chang, Meng-Chen Lee, Yi-Jhen Li

**Affiliations:** 1 Department of Health Services Administration, China Medical University, No. 91, Xueshi Rd., North Dist, Taichung City, 40402 Taiwan; 2 Department of Psychiatry, Changhua Christian Hospital, 135 Nanxiao St., Changhua City, Changhua County 50006 Taiwan; 3 Department of Medical Affairs, Landseed International Hospital, No. 77, Guangtai Rd., Pingzhen Dist, Taoyuan City, 32449 Taiwan; 4 Department of Health Services Policy and Management, University of South Carolina, 915 Greene St, Columbia, SC 29208 USA

**Keywords:** Category of mental disorders, Mortality, Medical utilization, Potential years of life loss, Expenditure survival ratio

## Abstract

**Background:**

The decreased life expectancy and care costs of mental disorders could be enormous. However, research that compares mortality and utilization concurrently across the major category of mental disorders is absent. This study investigated all-cause mortality and medical utilization among patients with and without mental disorders, with an emphasis on identifying the psychiatric category of high mortality and low medical utilization.

**Methods:**

A total of 570,250 individuals identified from the 2002–2013 Taiwan National Health Insurance Reearch Database consistuted 285,125 psychiatric patients and 285,125 non-psychiatric peers through 1:1 dual propensity score matching (PSM). The expenditure survival ratio (ESR) was proposed to indicate potential utilization shortage. The category of mental disorders and 13 covariates were analyzed using the Cox proportional hazard model and general linear model (GLM) through SAS 9.4.

**Results:**

PSM analyses indicated that mortality and total medical expenditures per capita were both significantly higher in psychiatric patients than those in non-psychiatric patients (all *P* <.0.0001). Patients with substance use disorders were reported having the youngest ages at diagnosis and at death, with the highest 25.64 of potential years of life loss (YPLL) and relevant 2904.89 of ESR. Adjusted Cox model and GLM results indicated that, compared with anxiety disorders, affective disorders and substance use disorders were significantly associated with higher mortality (HR = 1.246 and 1.064, respectively; all *P* < 0.05); schizophrenia was significantly associated with higher total medical expenditures per capita (*P* < 0.0001). Thirteen additional factors were significantly associated with mortality or utilization (all *P* < 0.05).

**Conclusion:**

Substance use disorders are the category of highest YPLL but notably in insufficient utilization. Health care utilization in patients with substance use disorders should be augmented timely after the diagnosis, especially toward home and community care. The factors related to mortality and utilization identified by this study merit clinical attention.

## Background

Mental disorders are leading causes of life years with disability worldwide [1]. The high impact on life expectancy of a certain category of mental disorders has been reported by previous studies, such as affective disorders [2, 3], substance use disorders [4], and schizophrenia [1, 5, 6]. The life impacts of such disease require substantial attention. Previous studies have indicated that mental disorders are highly contributed to the death causes of accident and self- inflicted injury [7–9]. According to prior research of mortality in patients with mental disorders, overall survival time after the diagnosis was 36.2 years [10]; average life expectancy (LE) and potential years of life loss (YPLL) among psychiatric patients was 55.5 years and 17.7 years, respectively [11, 12]. Nevertheless, age at the diagnosis of mental disorders highly impacts on the survival time. The survival time was greatly shortened to only 1.32 years among people who first experienced mental illness in age higher than 60 years [13]. Therefore, study of life impacts including survival time, mortality, death age, and YPLL substantiates the necessity of investigation on age at diagnosis.

Mental illness is debilitating because of functional impairment. The health care for psychiatric patients is rather challenging. Prior research has documented that health care expenditures on patients with mental disorders were higher than those on the general population [14]. Average medical expenditure per capita of psychiatric patients and non-psychiatric patients were reported to be 12,358 USD and 7927 USD, respectively [15]. Specifically, the average inpatient expenditure per capita in psychiatric patients was double of that in general public [16]; hospital length of stay in patients with depression disorder was also higher than non-depressive patients [17, 18]. Consequently, patients with mental disorders were likely to become high utilizers of medical care [19]. Use of emergency services, mental health assessment, and psychotherapy specific to psychiatric patients could lead to additional medical expenditures [20, 21].

Severe psychiatric patients with certain characteristics might be related with high mortality and medical expenditures. Previous studies have indicated that dual diagnosis (co-occurring disorders) and involuntary hospitalization, two indications of high psychiatric severity, were linked to a high risk of death [22] and high medical utilization [23]. Moreover, medical comorbidity, such as diabetes and cancer, could lead to higher mortality and medical utilization [24–28]. According to the existing literature, various medications have been linked to high mortality and expenditures, such as haloperidol, tricyclic antidepressants, and monoamine oxidase inhibitors [29, 30]. Overall, clinical severity in this study includes psychiatry-specific indications, namely dual diagnoses and voluntary hospitalization, and other medical characteristics, including comorbidity and medication. The aforementioned four medical characteristics should be included in the analysis.

Most of the population-based studies examined life outcomes between psychiatric and non-psychiatric patients without using a matching method [31–33]. Information on average age at diagnosis, LE, and YPLL for various categories of mental disorders is also lacking. Most psychiatric studies investigated one single type of disorder and reported on either mortality or medical expenditure [18]. Lack of research that investigates both mortality and expenditures using a paired comparison justifies this study. Out of a complete spectrum of mental disorders, which psychiatric category is linked to the high risk of premature death and low medical utilization is obscure. Therefore, the present study investigated the mortality and medical expenditures of the major category of mental disorders by using numerous factors. A certain category of mental disorders with relatively high YPLL and low utilization would be identified.

## Methods

### Hypothesis and research design

This study hypothesized that mortality and medical utilization were associated with various factors including health and medication characteristics, demographic characteristics, provider characteristics, and geographic characteristics. The presence and category of mental disorders were considered as the key factors. A longitudinal retrospective population-based design was used to examine the hypothesis by analyzing nationwide data of psychiatric patients versus matched non-psychiatric patients in Taiwan. The current study was reviewed and approved by the Research Ethics Committee at China Medical University and Hospital, Taichung, Taiwan, under no. CMUH105-REC3–016.

### Data source and study sample

The Taiwan National Health Insurance (NHI) program was launched in 1995 and covers more than 99.7% of 23.74 million people in Taiwan [1]. Both grants funded by China Medical University and the Ministry of Science and Technology provided support to obtain the study data, which were retrieved from the National Health Insurance Research Database (NHIRD), which contains the registration files and medical claims data including ambulatory and inpatient care for one million randomly sampled NHI beneficiaries from 2002 to 2013.

The diagnostic codes 290.x–319.x of the International Classification of Diseases, Ninth Revision, Clinical Modification (ICD-9-CM) were used to identify psychiatric patients. Patients with only one time of psychiatric diagnosis were excluded from the study to ensure a reliable identification of cases. Furthermore, to ascertain that study patients were newly diagnosed with mental disorders, those diagnosed with mental disorders in 2002 were excluded from the study. Consequently, 285,125 patients with mental disorders (study group) were matched with 285,125 counterparts without mental disorders (control group) at a 1:1 ratio according to a propensity score [34]. The matched variables included gender, age, comorbidity, urbanization level, and index date of diagnosis. After the use of the propensity score matching method, the selection bias was reduced and the similarity between the two groups was substantially enhanced [2–4].

### Variables

The outcome variables included all-cause death and medical utilization, both of which were observed in the period of 2003 to 2013. Death was defined dichotomously, whereas medical utilization was represented by a continuous measure of total medical expenditures per capita (in USD; exchange rate of 1 USD to 30 TWD as of November 6, 2019) comprising ambulatory and inpatient claims. The two major independent variables were the presence of mental disorders and the category of mental disorders. The presence of mental disorders was defined as claims with main diagnosis codes of 290.x–319.x [35]. Based on previous studies and frequency distributions, patients with mental disorders were classified into the following five categories: affective disorders (296.xx), anxiety disorders (300.xx, 308.3, and 309.81), substance use disorders (303.xx, 304.xx, and 305.xx), schizophrenia (295.xx), and other (the remaining codes) [36–38]. The category of mental disorders was determined on the diagnosis code of a mental disorder that occurred first (with a 1-year observation window of non-mental disorder claims) in 2003 and had at least two mental disorder claims within 6 months of first occurrence for a patient to ensure a reliable classification.

In addition to the presence of mental disorders and the category of mental disorders, based on the existing studies, the present research included the following four groups of characteristics that were possibly related to mortality and utilization among patients with mental disorders: 1. Health and medication characteristics: multiple diagnoses [22], involuntary hospitalization [23], comorbidity [28], and use of psychiatric medication [30]; 2. Demographic characteristics: gender [5], age [6], occupation [39], and premium-based monthly salary [40]; 3. Provider characteristics: level of hospital [41], ownership of hospital [41], and seniority of care psychiatrist [42]; 4. Geographic characteristics: region and urbanization level [43]. Among the five categories of mental disorders defined in this study, when a patient underwent more than one category during the observation period of 2003 to 2013, multiple diagnoses would be coded present; otherwise, absent. Comorbidity was measured using the Charlson comorbidity index (CCI) [44], a frequently used instrument in clinical research. Psychiatric medications were categorized from frequently used drugs in the treatments for patients with mental disorders. The seniority of major care psychiatrist for a patient in this study was classified into four levels on the basis of frequency distribution. Urbanization level was graded on a seven-level scale, with levels 1 and 7 indicating the highest and lowest urbanization levels, respectively. The rest of the variables were defined according to the official classifications of the registry for NHI beneficiaries and the medical claims. All the 15 independent variables were measured in a categorical or an ordinal level.

### Statistical analysis

The statistical methods in this study included the chi-square test, survival analysis, and general linear model (GLM). The chi-squared test was used to report the prevalence rates of all-cause death among patients with mental disorders in bivariate analysis. Cox proportional hazard model was used to estimate the risks of all-cause death during the 11-year observation period, with the adjusted hazard ratio (HR) being reported. GLM was selected to test the mean differences in total medical expenditures per capita because of its capacity for the estimation of means (least squares means) for each level of the independent variables when adjusting all other covariates [45]. Furthermore, derived from the concept of survival ratio reported by a previous study [46], a standardized measure, expenditure survival ratio (ESR) was proposed to represent medical expenditures invested per survival time. When the medical expenditure is similar, shorter survival time entails larger ESR, which potentially implying the effect of insufficient utilization on life outcome. Moreover, collinearity diagnostics was conducted using indices including tolerance and variance inflation. Data were analyzed using SAS Version 9.4 (SAS Institute, Inc., Cary, North Carolina).

## Results

The result of the 1:1 propensity score matching indicated no differences in the five matched variables, including gender, age, comorbidity, urbanization level, and index date of diagnosis, between patients with and without mental disorders (all *P* = 1), confirming that the two groups (285,125 versus 285,125, Table 1) were eligible for the comparisons. Furthermore, this study did not detect significant signs of collinearity. Propensity-matched analysis indicated that, after adjustment for all other covariates, the odds of all-cause death were significantly higher in patients with mental disorders than in those without mental disorders (HR = 1.150, 95% CI = 1.127–1.175, *P* < 0.0001). The estimated mean of total medical expenditures per capita in patients with mental disorders was also significantly higher than that in patients without such disorders (18,455.43 versus 14,248.66, *P* < 0.0001).

**Table 1:** Propensity score-matched comparisons of mortality and total medical expenditures per capita between patients with and without mental disorders, controlling for all other variables (Cox proportional hazard model and GLM, *N* = 570,250)

Variables	n	%	Death		***P***-value	Mean of total medical expenditures (USD)	***P***-value
			Adjusted HR	95% CI			
Mental disorders					< 0.0001*		< 0.0001*
Present	285,125	50.00	1.150	1.127–1.175		18,455.43	
Absent	285,125	50.00	–	–		14,248.66	

* *P* < 0.05

*HR* hazard ratio

Other variables in the models included: comorbidity, gender, age, occupation, premium-based monthly salary, level of hospital, ownership of hospital, seniority of care physician, region, and urbanization level.

The chi-square test results indicated that the category of mental disorders was significantly associated with the risk of death (Table 2, *P* < 0.0001). Among the five psychiatric categories, the mortality was higher in schizophrenia and affective disorders (11.67 and 10.67%, respectively). All other 13 independent variables were significantly associated with the mortality of psychiatric patients (all *P* < 0.05). Specifically, other health and medication characteristics significantly associated with a higher likelihood of death included the presence of multiple diagnoses (8.73%), the presence of involuntary hospitalization (12.89%), CCI ≥ 2 (29.40%), and use of multiple categories of psychiatric medication (13.89%).

**Table 2:** Mortality of psychiatric patients, by the category of mental disorders and other characteristics (Chi-square test, *N* = 285,125)

Variables	Frequency	%	Death		No death		χ^**2**^ ***P***-value
			n_**1**_	%	n_**2**_	%	
			23,136	8.11	261,989	91.89	
**Health and Medication Conditions**							
*Category of mental disorders*							< 0.0001*
Affective disorders	17,608	6.18	1879	10.67	15,729	89.33	
Anxiety disorders	136,642	47.92	8636	6.32	128,006	93.68	
Substance use disorders	22,830	8.01	1983	8.69	20,847	91.31	
Schizophrenia	1662	0.58	194	11.67	1468	88.33	
Other disorders	106,383	37.31	10,444	9.82	95,939	90.18	
*Multiple diagnoses*							< 0.0001*
Present	95,345	33.44	8320	8.73	87,025	91.27	
Absent	189,780	66.56	14,816	7.81	174,964	92.19	
*Involuntary hospitalization*							0.0051*
Present	256	0.09	33	12.89	233	87.11	
Absent	284,869	99.91	23,103	8.11	261,766	91.89	
*Comorbidity (CCI)*							< 0.0001*
0	182,941	64.16	7142	3.90	175,799	96.10	
1	89,485	31.38	12,261	13.70	77,224	86.30	
≥ 2	12,699	4.45	3733	29.40	8966	70.60	
*Use of psychiatric medication*							< 0.0001*
Mood stabilizer	95,758	33.58	8719	9.11	87,039	90.89	
Antidepressants	9078	3.18	605	6.66	8473	93.34	
Antipsychotics	8269	2.90	956	11.56	7313	88.44	
Multiple categories	47,170	16.54	6550	13.89	40,620	86.11	
None	124,850	43.79	6306	5.05	118,544	94.95	
**Demographics**							
*Gender*							< 0.0001*
Male	131,097	45.98	13,587	10.36	117,510	89.64	
Female	154,027	54.02	9549	6.20	144,478	93.80	
*Age (years)*							< 0.0001*
< 19	222,77	7.81	142	0.64	22,135	99.36	
20–34	50,083	17.57	943	1.88	49,140	98.12	
35–49	72,599	25.46	2546	3.51	70,053	96.49	
50–64	75,812	26.59	3463	4.57	72,349	95.43	
≥ 65	64,354	22.57	16,042	24.93	48,312	75.07	
*Occupation*^a^							< 0.0001*
First category	152,681	53.55	9215	6.04	143,466	93.96	
Second category	63,142	22.15	3736	5.92	59,406	94.08	
Third category	40,454	14.19	6170	15.25	34,284	84.75	
Fourth category	1078	0.38	67	6.22	1011	93.78	
Fifth category	1228	0.43	217	17.67	1011	82.33	
Sixth category	26,542	9.31	3731	14.06	22,811	85.94	
*Premium-based monthly salary (USD)*							< 0.0001*
≤ 576	163,453	57.33	12,014	7.35	151,439	92.65	
576–760	77,404	27.15	8891	11.49	68,513	88.51	
760–960	10,790	3.78	566	5.25	10,224	94.75	
960–1210	11,801	4.14	714	6.05	11,087	93.95	
1210–1526	10,177	3.57	513	5.04	9664	94.96	
> 1526	11,500	4.03	438	3.81	11,062	96.19	
**Provider Features**							
*Level of hospital*							< 0.0001*
Medical center	45,470	17.11	4131	9.09	41,339	90.91	
Regional hospital	70,237	26.42	6875	9.79	63,362	90.21	
District hospital	34,245	12.88	4307	12.58	29,938	87.42	
Clinic	115,852	43.59	6154	5.31	109,698	94.69	
*Ownership of Hospital*							< 0.0001*
Public	51,920	18.26	5742	11.06	46,178	88.94	
Private	157,478	55.37	10,565	6.71	146,913	93.29	
Consortium	67,277	23.66	5809	8.63	61,468	91.37	
Association	7733	2.72	675	8.73	7058	91.27	
*Psychiatrist’s year of practice*							< 0.0001*
≤ 5	74,789	26.23	4469	5.98	70,320	94.02	
6–10	103,619	36.34	8545	8.25	95,074	91.75	
11–15	49,063	17.21	4864	9.91	44,199	90.09	
≥ 16	57,654	20.22	5258	9.12	52,396	90.88	
**Geographic Characteristics**							
*Region*							< 0.0001*
Taipei	102,630	36.25	7279	7.09	95,351	92.91	
Northern	34,842	12.30	2854	8.19	91,988	91.81	
Central	54,734	19.33	4343	7.93	50,391	92.07	
Southern	40,425	14.28	3837	9.49	36,588	90.51	
Southeast	43,345	15.31	3865	8.92	39,840	91.08	
Eastern	7178	2.54	776	10.81	6402	89.19	
*Urbanization level*							< 0.0001*
Level 1 (Highest)	84,967	30.20	5635	6.63	79,332	93.37	
Level 2	82,327	29.26	6127	7.44	76,200	92.56	
Level 3	47,269	16.80	3758	7.95	43,511	92.05	
Level 4	39,430	14.02	3884	9.85	35,546	90.15	
Level 5	7076	2.52	870	12.30	6206	87.70	
Level 6	11,050	3.93	1453	13.15	9597	86.85	
Level 7 (Lowest)	9214	3.28	1096	11.89	8118	88.11	

^a^ First category includes private employees and public servants; Second category includes labor union members; Third category includes farmers and fishermen; Fourth category includes soldiers; Fifth category includes social security insureds; Sixth category includes veterans and religious group members

Table 3 lists the means for age at diagnosis of mental disorders, survival time, age at death, YPLL, and ESR, respectively, for the five categories of psychiatric patients who died in the observation period of 2003 to 2013. Among the five categories, patients with substance use disorders experienced the disease and died both earliest at the age of 51.38 and 54.38 years, respectively. Accordingly, substance use disorders were reported with the highest YPLL of 25.64 years, the ESR for substance use disorders was 2904.89, which is in concordance with the YPLL results. The GLM test results indicated significant differences in ESR between the categories of mental disorders (*P* < 0.0001). Schizophrenia and anxiety disorders have the highest and lowest average ESRs of 12,183.39 and 1236.34 among the five categories of mental disorders.

**Table 3:** Average potential years of life loss and expenditure survival ratio in patients with mental disorders, by the category of mental disorders (Descriptive statistics and GLM, *N* = 23,136)

Category of mental disorders	Age at diagnosis	Survival time	Age at death	YPLL	Expenditure survival ratio
Affective disorders	61.15	2.72	63.87	16.15	5411.26
Anxiety disorders	63.91	3.32	67.23	12.79	1236.34
Substance use disorders	51.38	3.00	54.38	25.64	2904.89
Schizophrenia	55.70	2.90	58.60	21.42	12,183.39
Other disorders	71.90	2.60	74.50	5.52	2307.80

YPLL was calculated on the reference age of 80.02 years (LE) for the Taiwanese population in 2013.

Expenditure survival ratio was estimated using multivariate GLM (*P* < 0.0001).

Table 4 presents the results of multivariate analyses for all-cause mortality and total medical expenditures per capita among patients with mental disorders. Anxiety disorders were used as the reference group for the category of mental disorders in the Cox model because of the lowest death rate (6.32%) among the categories. The adjusted Cox model results indicated that the category of mental disorders was significantly associated with the risk of death (all *P* < 0.05). Compared with patients with anxiety disorders, patients with affective disorders, substance use disorders, and other mental disorders were more likely to die (HR = 1.246, 1.064, and 1.233, respectively). When all other variables were constant, the other significant factors related to the risk of death among patients with mental disorders included multiple diagnoses, comorbidity, use of psychiatric medication, gender, age, occupation, premium-based monthly salary, level of hospital, ownership of hospital, seniority of care psychiatrist, region, and urbanization level (all *P* < 0.05). The high risk of death was associated with the following main characteristics: the absence of multiple diagnoses, CCI ≥ 2, use of antipsychotics, male, high ages, social security insureds, income between $760 and $960, regional hospitals, association-owned hospitals, psychiatrist seniority ≤10 years, southern and eastern regions, and lowest level of urbanization. Furthermore, information regarding death per 1000 person-days was listed in Table 4. Among the five categories of mental disorders, schizophrenia was associated with the highest death rate per 1000 person-days (*P* = 0.0685). Moreover, after adjustment for all other covariates, the GLM test results indicated significant means differences in total medical expenditures per capita between the categories of mental disorders (*P* < 0.0001). Schizophrenia was ranked the highest in total medical expenditures per capita with the estimation of USD 29,748.05. Ten other factors associated with mean differences in the medical expenditures included multiple diagnoses, involuntary hospitalization, use of psychiatric medication, gender, age, occupation, premium-based monthly salary, level of hospital, ownership of hospital, and seniority of care psychiatrist (all *P* < 0.05). Other characteristics associated with high averaged total medical expenditures per capita included the presence of multiple diagnoses, the presence of involuntary hospitalization, use of multiple categories of psychiatric medication, male, age ≥ 65, social security insureds, income ≤576, medical centers, public hospitals, and seniority of care psychiatrist ≥16 years.

**Table 4:** Association between the category of mental disorders, mortality, and total medical expenditures per capita, controlling for all other variables (Cox proportional hazard model and GLM, *N* = 285,125)

Variables	Death per 1000 person-days	Death		***P***-value	Mean of total medical expenditures (USD)	***P***-value
		Adjusted HR	95% CI			
**Health and Medication Conditions**						
*Category of mental disorders*						< 0.0001*
Affective disorders	0.0617	1.246	1.180–1.316	< 0.0001*	15,791.06	
Anxiety disorders	0.0335	–	–	–	12,618.92	
Substance use disorders	0.0500	1.064	1.009–1.122	0.0231*	13,307.55	
Schizophrenia	0.0685	1.072	0.923–1.245	0.3646	29,748.05	
Other disorders	0.0546	1.233	1.195–1.271	< 0.0001*	13,016.03	
*Multiple diagnoses*						< 0.0001*
Present	0.0488	–	–	–	17,275.36	
Absent	0.0421	1.095	1.062–1.130	< 0.0001*	16,517.29	
*Involuntary hospitalization*						< 0.0001*
Present	0.0892	–	–	–	28,345.00	
Absent	0.0443	1.417	0.988–2.032	0.0579	5447.64	
*Comorbidity (CCI)*						0.4944
0	0.0212	–	–	–	16,887.48	
1	0.0744	1.004	0.970–1.040	0.8134	16,869.13	
≥ 2	0.1825	1.178	1.126–1.233	< 0.0001*	16,932.35	
*Use of psychiatric medication*						< 0.0001*
Mood stabilizer	0.0503	1.061	1.025–1.098	0.0007*	16,743.08	
Antidepressants	0.0365	1.057	0.968–1.153	0.2169	16,357.20	
Antipsychotics	0.0579	1.237	1.152–1.330	< 0.0001*	16,591.78	
Multiple categories	0.0763	–	–	–	18,354.09	
None	0.0274	1.213	1.166–1.261	< 0.0001*	16,435.46	
**Demographics**						
*Gender*						< 0.0001*
Male	0.0578	1.039	1.009–1.069	0.0104*	17,058.96	
Female	0.0333	–	–	–	16,733.68	
*Age (years)*						< 0.0001*
< 19	0.0035	–	–	–	16,905.26	
20–34	0.0105	1.760	1.460–2.122	< 0.0001*	16,768.65	
35–49	0.0187	2.143	1.789–2.567	< 0.0001*	16,892.76	
50–64	0.0238	1.915	1.598–2.294	< 0.0001*	16,847.92	
≥ 65	0.1458	1.809	1.514–2.162	< 0.0001*	17,067.00	
*Occupation*^a^						< 0.0001*
First category	0.0327	1.073	1.021–1.127	0.0053*	16,757.76	
Second category	0.0318	–	–	–	16,719.60	
Third category	0.0847	1.066	1.011–1.124	0.0180*	16,749.59	
Fourth category	0.0338	1.058	0.817–1.371	0.6670	16,464.26	
Fifth category	0.1031	1.193	1.029–1.382	0.0190*	17,809.09	
Sixth category	0.0814	1.065	1.003–1.130	0.0400*	16,877.63	
*Premium-based monthly salary (USD)*						< 0.0001*
≤ 576	0.0407	1.095	0.987–1.214	0.0857	17,166.44	
576–760	0.0622	1.079	0.967–1.205	0.1755	17,050.43	
760–960	0.0277	1.185	1.038–1.352	0.0119*	16,888.68	
960–1210	0.0322	1.064	0.938–1.208	0.3322	16,879.77	
1210–1526	0.0264	1.024	0.895–1.171	0.7319	16,713.87	
> 1526	0.0198	–	–	–	16,678.74	
**Provider Features**						
*Level of hospital*						< 0.0001*
Medical center	0.0478	1.123	1.068–1.181	< 0.0001*	17,468.25	
Regional hospital	0.0550	1.200	1.146–1.256	< 0.0001*	17,110.40	
District hospital	0.0644	1.101	1.056–1.148	< 0.0001*	16,871.55	
Clinic	0.0295	–	–	–	16,135.08	
*Ownership of Hospital*						< 0.0001*
Public	0.0625	1.013	0.972–1.056	0.5297	17,341.45	
Private	0.0359	–	–	–	16,581.21	
Consortium	0.0464	1.012	0.968–1.058	0.6082	16,605.31	
Association	0.0669	1.139	1.044–1.243	0.0035*	17,057.32	
*Psychiatrist’s year of practice*						< 0.0001*
≤ 5	0.0484	1.709	1.629–1.793	< 0.0001*	16,838.19	
6–10	0.0436	1.237	1.193–1.283	< 0.0001*	16,843.31	
11–15	0.0445	1.020	0.980–1.062	0.3206	16,938.61	
≥ 16	0.0423	–	–	–	16,965.18	
**Geographic Characteristics**						
*Region*						0.1356
Taipei	0.0388	–	–	–	16,906.41	
Northern	0.0455	1.009	0.959–1.062	0.7265	16,897.61	
Central	0.0433	1.028	0.982–1.076	0.2401	16,819.57	
Southern	0.0516	1.087	1.036–1.142	0.0008*	16,891.88	
Southeast	0.0480	1.014	0.970–1.061	0.5367	16,878.81	
Eastern	0.0617	1.141	1.043–1.247	0.0038*	16,983.65	
*Urbanization level*						0.7318
Level 1 (Highest)	0.0361	1.063	0.976–1.158	0.1597	16,906.04	
Level 2	0.0405	1.080	0.996–1.171	0.0636	16,912.38	
Level 3	0.0435	1.069	0.984–1.161	0.1131	16,921.85	
Level 4	0.0541	1.050	0.970–1.136	0.2301	16,851.65	
Level 5	0.0688	–	–	–	16,928.21	
Level 6	0.0730	1.081	0.989–1.182	0.0855	16,872.15	
Level 7 (Lowest)	0.0645	1.114	1.013–1.224	0.0264*	16,881.97	

* *P* < 0.05

*HR* hazard ratio

^a^ First category includes private employees and public servants; Second category includes labor union members; Third category includes farmers and fishermen; Fourth category includes soldiers; Fifth category includes social security insureds; Sixth category includes veterans and religious group members

## Discussion

### Mental disorders in higher mortality and utilization

Through PSM, the higher mortality and utilization in patients with mental disorders were identified, warranting more attention throughout the disease course. The high rate of all-cause deaths among patients with mental disorders could be engendered by the higher risk of suicide [47, 48] and road traffic accidents [49] because of potentially emotional or cognitive instability leading to unnatural deaths [9]. From the perspective of comorbidity, psychosomatic susceptibility and pathological changes might lead to the potential bidirectional relationship between mental disorders and cancer [50], thus increasing the mortality of this vulnerable population [51]. Furthermore, certain psychiatric medications that slow down metabolism could increase body weight and further contribute to the developments of endocrine disorders and cardiovascular diseases [52, 53]. The psychophysiological conditions of psychiatric patients are closely related to the subsequently high mortality and medical expenditure per capita determined. The finding that total medical expenditures per capita in patients with mental disorders were higher than those in general population is consistent with a previous study [14].

### Affective disorders and substance use disorders in higher mortality

This study identified that affective disorders, substance use disorders, and other disorders had higher mortality rate. Indicated by a previous study, the mortality of anxiety disorders was relatively low, compared with that of affective disorders [54]. However, the high rate of suicides resulted in the high mortality among patients with affective disorders [55], with the YPLL of 11 to 20 years reported by prior research [6]. Furthermore, substance use disorders might increase the risk of traffic accidents [56, 57]. Notably, instead of natural causes of death, severe driver injuries in patients with substance use disorders could lead to a loss of 25 years in LE [58] that mirrors the finding of YPLL at 25.64 years in this study. Physio-psychological impairment and decreased level of consciousness induced by substance use as well as poor compliance with treatments are highly life-threatening [59] and thus merit policy emphasis.

### Substance use disorders in higher YPLL but in lower utilization

**Fig. 1:**
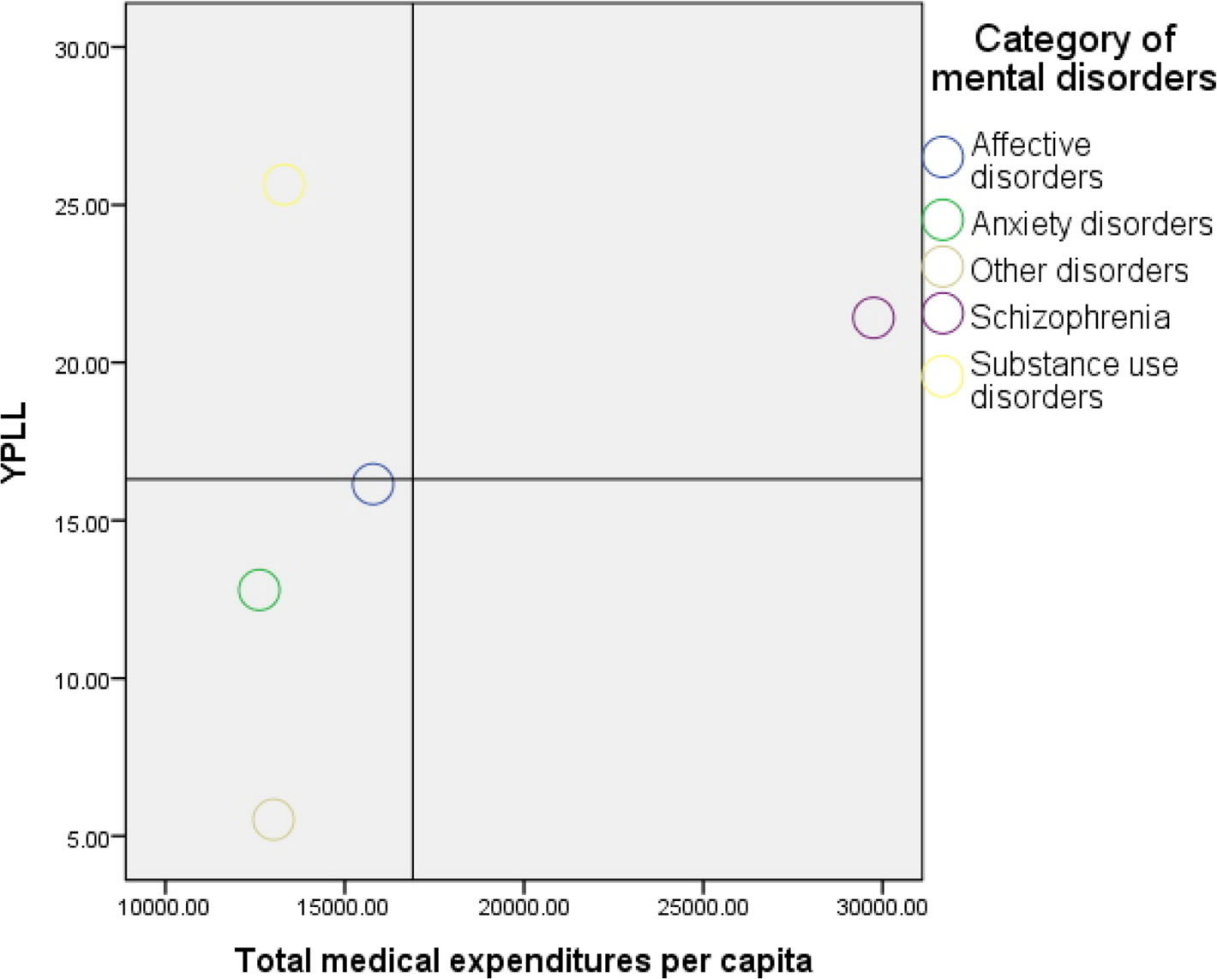
Scatterplots of YPLL and total medical expenditures per capita for the five categories of mental disorders

The averaged medical expenditures in patients with schizophrenia were estimated (USD. 29,748.05) to be almost double of those in patients with all other categories, which is similar to the finding of a previous study [60]. However, the mortality estimated in HR for patients with schizophrenia was not the highest among all the psychiatric patients. Furthermore, in a broader consideration of life impacts, ESR carries clinical and policy implications. Substance use disorders, with the earliest onset age and youngest death age, produced the notable ESR of 2904.89, which indicates a room for incremental utilization in terms of premature death. Figure 1 depicts the scatterplots by the two dimensions of YPLL and total medical expenditures per capita for the five categories of mental disorders. According to the classification in this graph, substance use disorders are the only category that falls into the quadrant of high YPLL and low medical expenditures. Substance use disorders are related to a variety of medical treatment, family, and social problems. In view of effects of substance use disorders on the family, effective interventions and mutual support programs involving engagement of the family members are crucial [61]. Therefore, in addition to acute care in hospitals, decision-makers should re-allocate health care resources into a non-acute health network involving home and community-based services (HCBS) where patients with substance use disorders reside to form a more comprehensive safeguard that should be launched timely after the diagnosis of substance use disorders.

### Other factors related to mortality and utilization merit medical attention

Other characteristics associated with high mortality among patients with mental disorders, such as CCI ≥ 2, use of antipsychotics, and low seniority of care psychiatrist, deserve more clinical attention and require further research. The improvement of life outcomes among patients with mental disorders should focus on early prevention for the risk of comorbid physical illnesses [62]. Moreover, the identified additional characteristics related to high medical expenditures are supported by the evaluated need component of the well-known health care utilization behavioral model [63], such as multiple diagnoses, involuntary hospitalization, use of multiple categories of psychiatric medication, and the lowest income bracket because of easy access to health care under NHI. Extant studies that investigated the various factors of medical expenditures are scarce. These findings on expenditures are valuable to the field of health services administration. Further scrutiny is required on the associations of low seniority–high mortality and high seniority–high utilization because the two links may intriguingly imply the necessity of human resources strategy concerning clinical performance. Overall, a majority of the related predictors performed differently in mortality and utilization.

The limitations of the current study stem from the NHIRD used. First, the NHIRD encompasses only government-reimbursed data, representing the majority of medical utilization. Self-provided medical items are not included in the database; thus, extrapolation of the study findings to other medical circumstances requires deliberation. Second, the NHIRD used does not contain data pertaining to education, marital status, and lifestyle, thus limiting further analysis in this study. Nevertheless, this study fully utilized all obtainable data for analysis. Finally, based on existing literature and frequency distributions, this study analyzed for the five major categories of mental disorders. Other category of mental disorders could not be further classified in this study. Future related research should concentrate on mortality and utilization for minor categories.

## Conclusions

Among the five categories of mental disorders, patients with schizophrenia faced the highest death rate and used medical resources the most. However, this study identified patients with substance use disorders as the vulnerable group of the highest YPLL but with substantially insufficient medical utilization, highlighting a noteworthy health disparity. Policy-makers should strengthen access to health care among patients with substance use disorders, especially HCBS, and intervene timely in the high-risk patients using the mortality related factors identified by this study. The present study suggests that life impacts and medical utilization should be investigated concurrently for a comprehensive analysis in order to improve the welfare of patients with mental disorders.

## Data Availability

The datasets used and/or analyzed during the current study are available from the corresponding author on reasonable request.

## Abbreviations

CCI: Charlson comorbidity index
CI: Confidence interval
ESR: Expenditure survival ratio
GLM: General linear model
HCBS: Home and community-based services
HR: Hazard ratio
ICD-9-CM: International Classification of Disease, Ninth Revision, Clinical Modification
LE: Life expectancy
NHI: National Health Insurance
NHIRD: National Health Insurance Research Database
YPLL: Years of life loss
